# Fault Detection and Isolation Methods in Subsea Observation Networks

**DOI:** 10.3390/s20185273

**Published:** 2020-09-15

**Authors:** Sa Xiao, Jiajie Yao, Yanhu Chen, Dejun Li, Feng Zhang, Yong Wu

**Affiliations:** State Key Laboratory of Fluid Power and Mechatronic Systems, Zhejiang University, Hangzhou 310027, China; sxiao@zju.edu.cn (S.X.); 21625146@zju.edu.cn (J.Y.); li_dejun@zju.edu.cn (D.L.); fengzh@zju.edu.cn (F.Z.); 11925110@zju.edu.cn (Y.W.)

**Keywords:** deep learning, fault detection, fault isolation, neural network, observation network

## Abstract

Subsea observation networks have gradually become the main means of deep-sea exploration. The reliability of the observation network is greatly affected by the severe undersea conditions. This study mainly focuses on theoretical research and the experimental platform verification of high-impedance and open-circuit fault detection for an underwater observation network. With the aid of deep learning, we perform the fault detection and prediction of the network operation. For the high-impedance and open-circuit fault detection of submarine cables, the entire system is modeled and simulated, and the voltage and current values of the operating nodes under different fault types are collected. Numerous calibrated data samples are supervised by a deep learning algorithm, and a fault location system model is built in the laboratory to verify the feasibility and superiority of the scheme. This paper also studies the fault isolation of the observation network, focusing on the communication protocol and the design of the fault isolation system. Experimental results verify the effectiveness of the proposed algorithm for the location and prediction of high-impedance and open-circuit faults, and the feasibility of the fault isolation system has also been verified. Moreover, the proposed methods greatly improve the reliability of undersea observation network systems.

## 1. Introduction

Cabled submarine observation networks are underwater monitoring networks formed by connecting many monitoring terminals/equipment distributed in the ocean through photoelectric composite cables. These networks are connected to the land transmission network grid and the communication network to realize the excellent span of land monitoring systems extending to the deep sea [1,2,3,4]. The reliable, safe, and stable power transmission of submarine observation networks is the key to ensuring that all underwater observation equipment can carry out scientific research and exploration work normally [5]. However, subsea observation networks, which are composed of many underwater photoelectric composite cables, electrical connectors, connecting equipment, and observation terminals, operate in deep sea and other harsh environments. Given their complex structure and poor working environment, power transmission systems are prone to failure, which affects the stability of power transmission. Submarine cable fault is an important factor that affects the reliability of submarine observation networks. In the case of ground faults of submarine cables, most of the current transmitted by shore stations flows through a short-circuit point, because the network transmits electric energy through a single line; consequently, the constant voltage power supply becomes paralyzed and stops immediately because the current exceeds the threshold value, and all the corresponding underwater connection boxes cannot be started normally; these conditions should be avoided as much as possible [6,7].

In recent years, most of the world’s undersea observation networks have carried out submarine cable fault location research. Most underwater fault diagnosis and location algorithms are based on the method proposed by North Eastern Pacific Time-Series Undersea Networked Experiment (NEPTUNE)-Canada, the first and the largest regional submarine observation network in the world with a constant voltage power system. Kevin and Chen Ching proposed a fault location algorithm. In this algorithm, the current and voltage values of known nodes are combined into a matrix Zmeas, and the voltage X of the branch point to be estimated, the topological structure matrix H, and the measurement error ε are combined with the admittance matrix. The weighted least squares method is used to address the heteroscedasticity problem caused by unsteady measurement errors. This algorithm is complex and requires a complete system topology matrix database. When the number of open-circuit faults increases, the matrix database also increases exponentially, thereby reducing the feasibility of the algorithm [8,9]. Lu Shuai and El Sharkawi also proposed a complete set of fault diagnosis and location algorithms for the topology errors and high-impedance faults of NEPTUNE. On the premise of the correct topology of the system, the admittance matrix of the system was constructed, and a series of cable faults (e.g., cable open circuit, high impedance, and cable short circuit) were effectively diagnosed by the error vector of the matrix operation [10,11]. Lu’s algorithm needs to combine the relevant data of the designed underwater branching device and junction box equipment, but it does not consider the huge impact of the Zener diode on the system model and node state equation, and there is a certain deviation in the actual diagnosis and positioning. Ting Chang of the University of Washington improved the diagnosis and location of the serious cable fault of Lu. Considering the existence of Zener diodes on backbone cables and on the premise of determining a ground fault section, the study used the principle of the shortest power supply line to establish and solve the nonlinear equations following Kirchhoff’s law of voltage and current and to determine the specific locations of the fault point [12,13]. There are many studies in which the neural network is applied to fault diagnosis: Ref. [14] proposed a deep learning method for multi-channel perceptual signals combining feature fusion; it is validated in the intelligent fault recognition of an automobile main decelerator. The deep structure of multi-channel sensor signal feature extraction and feature fusion can effectively identify the main decelerator fault mode, and the diagnostic accuracy can reach up to 95.84%. A novel fault diagnosis technology of rotating machinery based on deep learning is proposed in [15], the original three-axis accelerometer signal is input into a deep learning layer as a high-definition one-dimensional image, and the signal features are automatically extracted, which realizes the effective classification of different states of rotating machinery. Ref. [16] proposed a fault diagnosis method of an electromechanical system based on deep learning. The main advantages of this method are its easy application and high adaptability to available data. Ref. [17] proposed an integrated convolution neural network model for bearing fault detection. The framework of this model includes three branches of convolution neural network: one branch of multi-channel fusion convolutional neural network and two branches of one-dimensional convolutional neural network. In addition, a support vector machine integration strategy is used to fuse the results of multiple branches, which improves the generalization ability and robustness of the model. There are many studies on the fault location and isolation of terrestrial power grids all over the world: Ref. [18] uses wavelet coefficients of the current signal to identify fault direction. The fault directions identified in different positions in the system are combined to determine the fault line and isolate it. Ref. [19] proposed a new single-phase to ground fault location algorithm based on DERS (Distributed Energy Resources). It only needs fault information provided by the directional fault passage indicator and intelligent electronic device, and it does not need to know the status of the circuit breaker or the network topology. The proposed algorithm is only applicable to a radial network or network configuration.

Generally, the power and communication system of the land power grid are separated, while the communication system of the observation network is completely dependent on the power system. When the power system fails, the communication system also fails. Therefore, it is necessary to study the fault detection and isolation methods suitable for an undersea observation network.

## 2. Overview of the Subsea Observation Network

**Figure 1:**
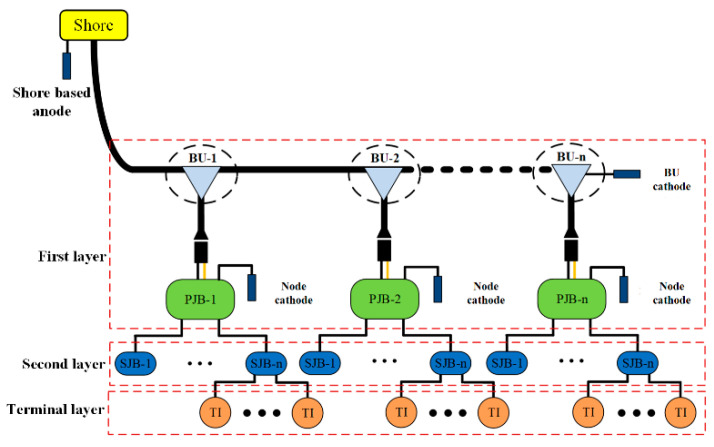
Overall system architecture of observation network.

The overall system architecture of the observation network is shown in Figure 1. The observation network in this work is composed of a power supply in the shore station (SS) and equipment in the first, second, and terminal layers. The first layer mainly includes an underwater photoelectric composite cable, underwater branch units (BUs), independent cathodes of BUs, and primary junction boxes (PJBs) connected in parallel to the backbone cable; each PJB has a corresponding node cathode. The first layer not only provides energy for the entire system but also accounts mainly for the high maintenance and repair costs. The second and terminal layers mainly include the secondary junction boxes (SJBs) and various terminal instruments.

Each PJB is connected to the backbone cable of the submarine observation network through a BU. High-voltage energy is gradually distributed to each terminal instrument through the PJB and SJB. The second and terminal layers have their own energy and communication management systems, which can isolate and work independently under various operation failures [1,4].

## 3. Fault Detection Methods

### 3.1. System Structure

**Figure 2:**
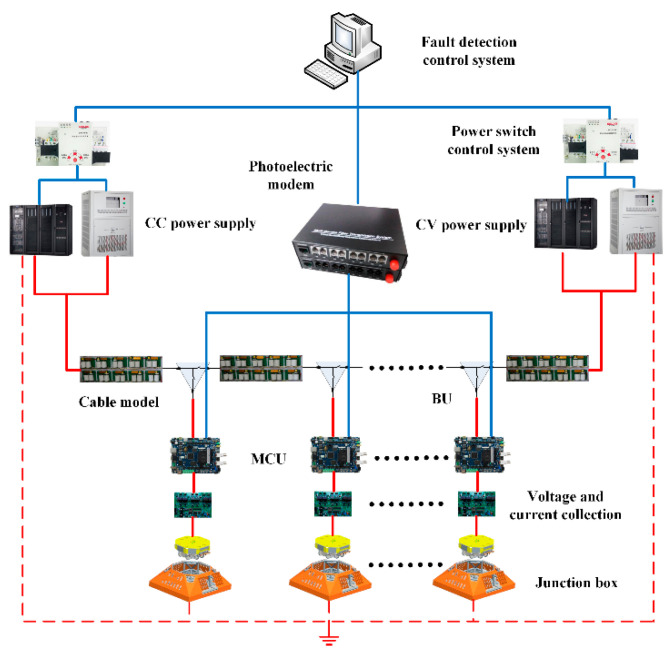
Diagram of fault detection.

This paper mainly focuses on the fault diagnosis of submarine cables, which can be divided into high-impedance faults, open-circuit faults, and short-circuit faults. High-impedance faults and open-circuit faults are common faults. Short-circuit faults are a product of high-impedance faults. If a high-impedance fault cannot be found and repaired in time, the damage degree of the submarine cable will be expanded, and the high-impedance fault will turn into a short-circuit fault, and the whole system will collapse. Therefore, monitoring high-impedance faults can greatly reduce the probability of short-circuit faults. The fault detection method proposed in this paper mainly aims at high-impedance and open-circuit faults. The fault detection system mainly monitors the voltage and current data of junction box nodes, uploads real-time signals to the shore-based power monitoring platform for data analysis and judgment through a microcontroller signal and photoelectric modem, and determines whether to use the power switching control system to start the fault isolation program according to the diagnosis results. The related hardware scheme is shown in Figure 2.

**Figure 3:**
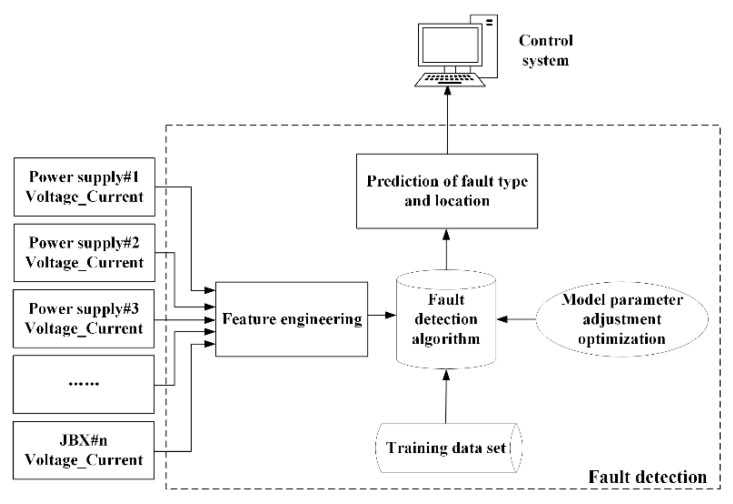
Flowchart of fault detection.

The entire fault detection algorithm is the control core of the entire platform. Deep neural network (DNN) is used to optimize and improve the accuracy of fault diagnosis and location [20,21]. Given the randomness and uncertainty of the location of cable faults, time limitations, and economic constraints, realistically simulating the large amount of data required in deep learning training is difficult. Therefore, the dataset is enhanced by the software modeling of the observation network. Deep learning uses a common DNN for model training based on supervised learning. It also sets the characteristics of the training data set x^(i)^ = {voltage and current data of power supply, voltage and current data of all junction boxes in a complex topology network} and the corresponding label y^(i)^ = {calibration value of corresponding fault type}. The TensorFlow (Tensorflow is a symbolic mathematical system based on data stream programming, which is widely used in the programming of various machine learning algorithms. It is developed and maintained by Google brain, a team of Google artificial intelligence in California, USA) deep learning computing framework is used for DNN training, and the cost reduction function is optimized for fault detection model training. The specific fault detection flowchart is shown in Figure 3.

(1) A system simulation model is built in the PSpice simulation software (Pspice is launched by Cadence in San Jose, CA, USA), and high-impedance faults are simulated at intervals by connecting a series of fault resistors at the fault point. Python is used to preprocess the simulation data (such as the normalization of feature combinations) to obtain the training set and test set data of the DNN.
(2) The TensorFlow deep learning framework is used to build a neural network predictive failure model and perform multiple iterative training optimizations for the training set characteristics. Meanwhile, the test set data are used to perform hyperparameter search tuning on the model to improve the model’s accuracy and generalizability. The trained fault detection model is saved locally to be used on real data.
(3) The voltage and current data of the shore base and junction box collected by the single-chip microcomputer are displayed in real time by the QT host computer (QT is a cross-platform C + + Graphical User Interface(GUI) application development framework developed bythe software company QT in Espoo, Finland in 1991. It can be used to develop both GUI programs and non-GUI programs, such as console tools and servers), and the collected related data are saved locally in text/csv format.
(4) The host computer software calls the Python (Python has become one of the most popular programming languages. It was founded by Guido van Rossum of Amsterdam, the Netherlands) interpreter to run the deep learning script and performs the same processing on the collected data according to the training data preprocessing steps. The combined features are used as input to the already trained deep learning model for prediction. The Qt host computer displays the prediction results on the graphical user interface in real time.
(5) If the system is predicted to have potential high-impedance and open-circuit faults, then the result can also be used as a drive signal for the fault isolation system to perform switching.

### 3.2. Supervised Learning Feature Engineering

**Figure 4:**
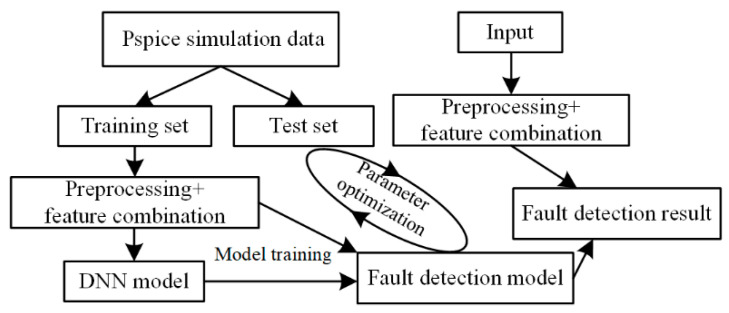
Fault detection process based on deep learning.

The current mainstream circuit simulation and design software mainly includes Multisim (Multisim is a windows based simulation tool developed by National Instruments (NI) Co., Ltd. in Austin, TX, USA), Saber (Saber is an electronic design automation software developed by Synopsys, in Mountain View, CA, USA), and PSpice. PSpice has a friendly graphical interface, simple operation, and excellent performance in circuit simulation, so we choose PSpice to simulate the circuit to obtain the dataset. In this work, a submarine observation network model with two shore base network topologies is established in PSpice, and the input voltage and current of each junction box are collected. After the prestandardized data processing, the deep learning input layer data are obtained. Given enough differences between each state feature, the fault detection model generated by the iterative training of the deep learning algorithm can accurately fit and distinguish the differences between systems. The fault detection process is shown in Figure 4.

**Figure 5:**
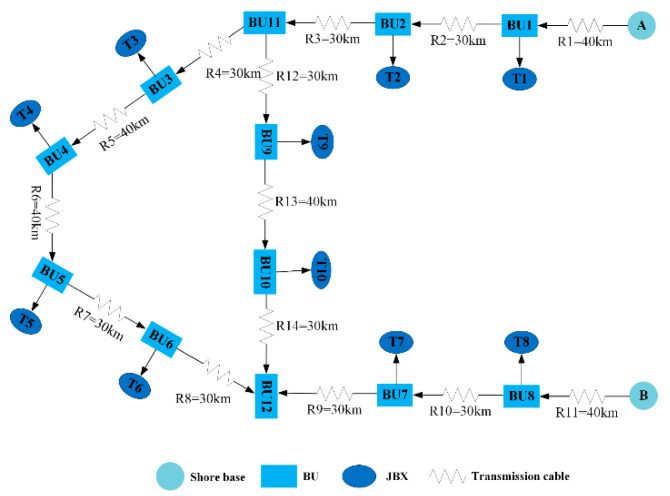
Schematic of an observation network with dual shore station mesh topology.

**Figure 6:**
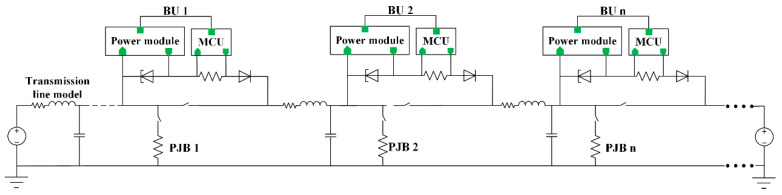
PSpice simulation model.

The network in PSpice (Figure 5 and Figure 6) consists of dual shore-based power supplies (A and B), 10 underwater nodes (T1–T10), 12 BUs (BU1–BU12), and 14 cable sections (R1–R14) and uses the combination of parasitic resistance, inductance, and capacitance to simulate the submarine cable. The neural network fault diagnosis method proposed in this paper is suitable for single shore station and dual shore station systems. A dual shore station system has better reliability than a single shore station system. When one shore power supply failure occurs, the other shore power supply can continue to provide power to ensure that the system does not crash. However, since the whole systems of single shore station and dual shore station systems are in different power states, in order to obtain the neural network model that can be applied to a single shore station system and dual shore station system respectively, it is necessary to obtain the training set of the corresponding system and conduct deep learning.

**Figure 7:**
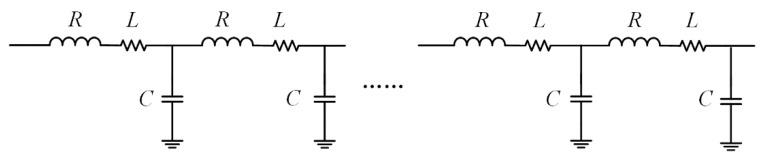
Lumped parameter cascade model of a transmission cable.

The lumped parameter model and distributed parameter model are two main transmission cable models [22]. The lumped parameter model is suitable for the steady-state calculation of a short distance transmission system. The distributed parameter model is accurate but computationally intensive. When considering the transient process of long-distance transmission cables, the lumped parameter cascade model showed in Figure 7 is generally used. After optimizing the segment length of a transmission cable, the lumped parameter cascade model can ensure the simulation accuracy and convergence speed. Ref. [4] proposed the parameters of a transmission cable: resistance R=1 Ω/km, inductance L=0.37 mH/km, and capacitance C=0.16 μF/km.

The simulation model mainly includes a shore-based power supply model, transmission cable model, BU model, and junction box model. Among them, the shore-based power supply is regarded as a DC voltage source due to its sufficient power. The transmission cable model adopts lumped parameter cascade model, and the specific parameters are determined according to the actual cable length. The BU model is determined according to the BU structure design. The junction box is a constant power load, and the specific model is determined according to [4].

Given the influence of temperature, damage degree, and seawater chemical characteristics, the ground resistance of high-impedance faults varies. Previous statistical data indicate that the ground resistance of high-impedance faults is generally in the range of thousands of ohms to hundreds of thousands of ohms. A series of different fault resistance values can be simulated at the same fault point in the simulation software, and the open-circuit fault in the simulation is obtained by turning off the BU.

The probability of multiple faults occurring simultaneously in a submarine observation network is considerably small. All the fault data in the simulation are based on the assumption that the system has a single fault in the network. The characteristics and the corresponding calibration values of the supervised learning training data adopted are shown in Table 1 and Table 2.

Each training sample has an input characteristic dimension ($R^{1*23}$), which is a continuous dataset that can be collected. The frequent fluctuation of the current value of the power supply when a fault occurs can be understood as a strong characteristic component that can be used to distinguish fault types. By using the product of two SS currents as a new feature, the identification degree of fault detection can be improved in the later model training. For each eigenvector ($x^{\left(i\right)}$), we manually calibrate the output calibration value ($y^{\left(i\right)}$) whose dimension is $R^{1*28}$. If the characteristic component ($y^{\left(i\right)}$) is 1, then the system is in a normal working state; otherwise, the system has a high-impedance or open-circuit fault. The 0/1 state value of the characteristic component ($y_{2\sim 13}{}^{\left(i\right)}$) indicates that the 12 BU switches have an open-circuit fault. The 0/1 state value of the characteristic component ($y_{14\sim 27}{}^{\left(i\right)}$) indicates that the 14 cable sections have a high-impedance fault. The sequence of the relevant BU switches and the cable sections is consistent with the network constructed in Figure 5. According to the previous assumption that only one fault occurs in the network at a time, only 1 of the 27 feature components can appear as 1, and the remaining feature components are equal to 0. The characteristic component ($y_{28}{}^{\left(i\right)}$) is valid only when any characteristic component of $y_{14\sim 27}{}^{\left(i\right)}$ is 1 (high-impedance faults occur in 14 sections of the cable). This value is a continuous value whose size does not exceed the maximum length of the faulty cable section and indicates the high-impedance fault location.

### 3.3. Training and Optimization of Fault Detection Model

The code implementation of the hidden layer of the neural unit mainly defines the function named add_layer shown in Algorithm A1 in Appendix A. The add_layer function mainly defines the weight variable ‘Weights’ and bias variable ‘biases’. Meanwhile, the two-dimensional weight and one-dimensional offset tensors of the hidden layer are initialized to be normal and constant for the convenient training and convergence of the neural network in the later stage. Then, operation ‘Wx_plus_b’ is defined to perform matrix operation on the output variables of the previous layer (input variables of the neural unit of this layer), the weight variables, and the bias variables. In a specific application scenario, we choose different activation functions, outputs = activation_function(Wx_plus_b), to deal with the calculation tensor nonlinearly and further increase the complexity of the system. The output of this hidden layer is the result calculated by this function and the input tensor of the neural unit of the next layer.

Compared with other activation functions such as sigmoid and tanh, when the input value of the relu activation function is greater than 0, the function gradient value is constant to 1, which greatly accelerates the training speed of the model. Without exponential operation, the convergence speed will be further accelerated. Therefore, the relu activation function is used in the model training of this research, which has an excellent training effect and can meet the training requirements of the diagnostic model well.

Combined with the fault detection feature engineering designed in Section 3.2, two cost functions are defined in the output layer of the DNN shown in Algorithm A2: the cross-entropy cost function is mainly used to evaluate the error degree of the softmax classification problem [23], and the cross-entropy cost function of the first 27 output features of y^(i)^ is calculated to get the similarity between the predicted value and the true value, which mainly solves the problem of fault classification. The cross-entropy loss can be calculated from:

(1) $$J\left(\theta \right)=-\frac{1}{m}\sum_{i=1}^{m}y^{\left(i\right)}\times \log(y^{(i{)}^{\prime }})+(1-y^{\left(i\right)})\times \log(1-y^{(i{)}^{\prime }})$$

The mean square deviation of the last output feature of y^(i)^ is calculated to measure the similarity of the regression problem and get the specific location of the predicted failure. The calculation formula of the mean square loss is:

(2) $$J\left(\theta \right)=\frac{\sum_{i=1}^{m}{\left(y^{\left(i\right)}-y^{(i{)}^{\prime }}\right)}^{2}}{m}$$

Finally, the two cost functions defined by feature engineering are weighted and summed to obtain the total cost function ‘loss’ of the system, which is used to minimize the global loss function to train the model. In this paper, the Adam optimizer with a better comprehensive performance is used to train the model iteratively [24]. Finally, the execution function ‘sess.run’ starts the adjustment of model parameters, and the loss function value is also decreasing.

Deep learning is end-to-end learning, and no theoretical support is given for the specific values of the neural network architecture and algorithm hyperparameters. Hyperparameter search is carried out continuously on the basis of personal experience to ensure that the prediction accuracy and generalization rate of the model meet the requirements. The accuracy index is the statistical result calculated from the training dataset that is aimed at measuring the ability of the model to fit the data. The generalization index is aimed at the test set without training to measure the model’s ability to predict unknown samples. In model training, the parameters must be adjusted continuously to optimize the training process. The relevant hyperparameters and corresponding initial values involved in the model are shown in Table 3. The proposed fault detection neural network consists of four hidden layers. By adjusting the model parameters, the number of neural units in each layer is determined to be 22, 20, 40, and 28, and the number of training iterations is 20,000. In total, 1064 sets of data are obtained from PSpice simulation software as the training set data of the neural network.

**Figure 8:**
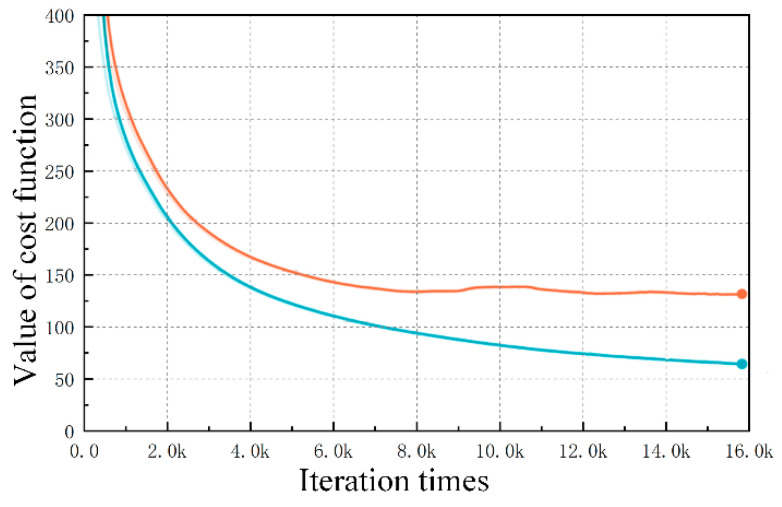
Curve of total cost function *Loss.*

Backpropagation (BP) is carried out with tf.train.AdamOptimizer, a built-in optimizer of TensorFlow, to calculate the partial derivative value of the cost function for all model parameter variables and optimize the model with the parameter update rate of the product of the learning rate and the corresponding partial derivative. To observe whether the optimization direction of the entire neural network is correct, we record the total cost function (Loss) of the training (blue curve) and test (orange curve) sets during the training process for every certain interval iteration (Figure 8).

The difference between the training set and the Bayes error rate (The Bayes error rate is the minimum error that can be achieved by inputting the existing feature set into any classifier. It can also be called minimum error.) is defined as bias, and the difference between the test and training sets is defined as variance. All parameters of the neural network are optimized to balance the bias and variance. According to Figure 8, the entire model training optimizes the training set, and the loss function value of the training set is smaller than that of the test set with the same data distribution. However, a difference of 50 remains between the loss value of the training set (blue curve) and 0 at the end of the training, indicating that the complexity of the current training model is not enough to fit the training set data of supervised learning and that the model suffers from high bias. In addition, the error between the loss value of the training and test sets increases with the number of training iterations. When the number of iterations is 16,000, the loss difference between the training and test sets is approximately 80. These data show the weakening generalizability of the fault detection model and its high variance problem.

The most common way to reduce variance is to add an additional term after the loss function. Two common additional terms are used: L1 regularization (ℓ1-norm) and L2 regularization (ℓ2-norm) [25]. L1 regularization refers to the sum of the absolute values of each element in weight vector W and is usually expressed as ||w||1; L2 regularization refers to the root of square sum of each element in weight vector w and is usually expressed as ||w||2. In the process of minimizing the cost function, the regularization term and the penalty function are added to the cost function for optimization to minimize the matrix parameter W value of the model. The final effect is equivalent to reducing the complexity of the model matrix and achieving a good trend for the generalized fitting data. In addition to regularization techniques, early stopping and dropout can be used to slow down the overfitting. The former continuously observes the loss curve of the test set. When the cost function increases with the number of iterations, the training must be stopped in advance. The latter randomly deletes the neural units of each layer according to a certain ratio. The equivalent effect is that the structure of the network becomes simple, thereby reducing the effect of overfitting. The fault detection model mainly uses the L2 regularization constant term and dropout technology to improve the generalizability.

To further reduce the bias between the predicted value of the training set and the label data, this work attempts to increase the numbers of hidden layers and neural units in each layer to increase the complexity of the model. However, the convergence slows as the network deepens. In severe cases, the gradient of the training parameters of the entire neural network disappears, and normal training cannot be performed. The BN (Batch Normalization) method solves the stoppage of model training. This technology adopts the idea of normalizing the mean value of the input layer data and constructs the input value of each neural layer of the hidden layer into a normal distribution with mean value γ and variance β through a normalization approach. In this way, the input value of the activation function always falls in the region of the nonlinear function with a large gradient, and the problem of gradient disappearance is avoided. The entire BN precedes the activation function shown in Algorithm A1, and the relevant calculation formula is expressed in the equations below.

(3) $$\mu_{B}=\frac{1}{m}\sum_{i=1}^{m}x_{i}$$

(4) $$\sigma_{B}^{2}=\frac{1}{m}\sum_{i=1}^{m}{\left(x_{i}-\mu_{B}\right)}^{2}$$

(5) $$\hat{x_{i}}=\frac{x_{i}-\mu_{B}}{\sqrt{\sigma_{B}^{2}+\varepsilon }}$$

(6) $$y_{i}=\gamma \times \hat{x_{i}}+\beta$$

In order to ensure that each layer of the neuron calculation model has a certain nonlinearity, after standardizing the matrix data, BN also sets the variance as scale (γ) and the mean value as shift (β). The core idea of the algorithm is to change the data distribution of each layer of the neural network forcibly to avoid the input value of the activation function appearing in the nonlinear interval, and the convergence speed of neural network is greatly accelerated without reducing the nonlinear complexity of the system.

Since the mean and variance fc_mean, fc_var of each layer cannot represent the data distribution of the whole training set, we must smooth the calculation results to standardize the available test data. In the Tensor Flow framework, the exponential weighted average operation function ‘ema’ is used to estimate the local mean value of variables, which makes the update of variables related to the historical value in a period of time. The batch standardization of each hidden layer can not only greatly improve the training speed, but also increase the classification effect. After BN processing, a large learning rate can be used in the training process without worrying about the non-convergence of the training such as oscillation, which can better train the complex neural network and to a certain extent prevent the over fitting of the training.

**Figure 9:**
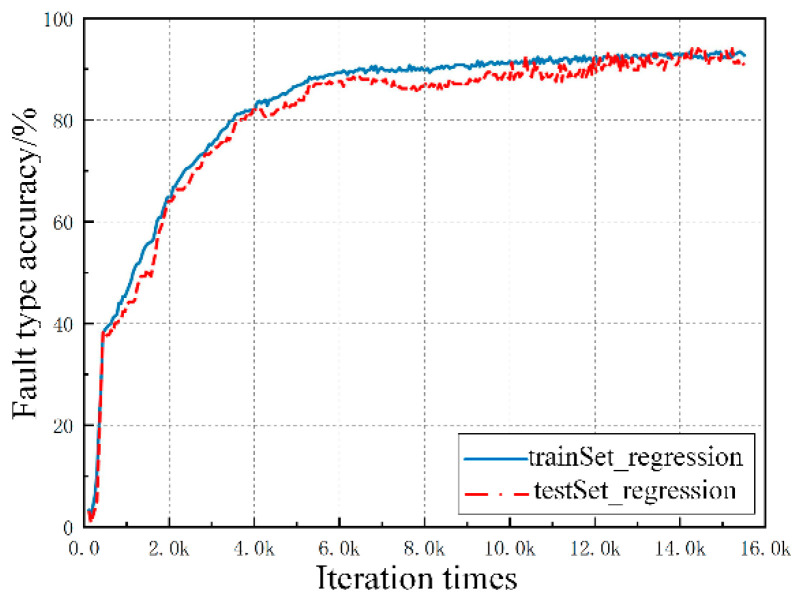
Prediction chart for fault type classification.

**Figure 10:**
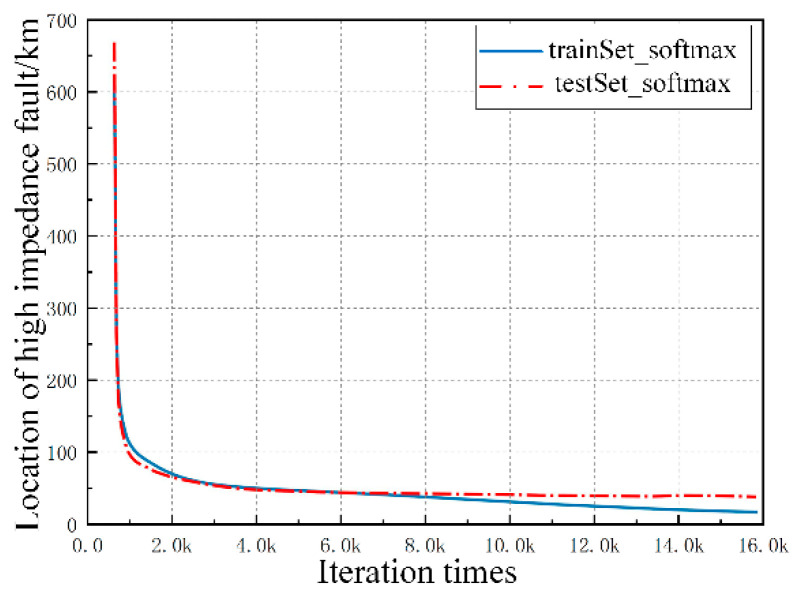
Regression prediction for fault location.

After a series of parameter adjustments and algorithm optimizations, the system fault detection model is completed. The test results are shown in Figure 9 and Figure 10. The blue curve represents the prediction accuracy of the training set, while the red curve represents the diagnosis results of the test set.

The above figure indicates that the prediction accuracy and generalizability of the fault detection model are further improved after the continuous optimization of the number of deep network layers, the number of neural units in each layer, the learning rate, the batch normalization, and the regularized dropout/L2 values.

Figure 9 describes the prediction accuracy of the multiclassification softmax problem with fault types. The diagnostic accuracy of the training and test sets gradually improves with the increase of the training times. The accuracy of the training set is always higher than that of the test set, and the accuracy error between them decreases. When the number of iterations reaches 16,000, the prediction accuracy of the fault detection model for high-impedance and open-circuit fault types reaches approximately 91%.

Figure 10 shows the prediction error diagram of the specific location of the high-impedance fault. The mean squared error of the fault location decreases with the increase of the number of training iterations. With the increase of iterations, the model keeps approaching the data law of training sets. At first, the accuracy will gradually increase with the number of iterations, but after a certain number of iterations, which is 8000 in this simulation, because of the excess of model expression ability, this neural network will learn some non-common features that can only meet the training sets (these are more of an accidental feature, not applicable to the test sets, which means there is an over fitting), which will lead to the decline of test accuracy. Therefore, in the over-fitting state, the accuracy will first increase and then decrease with the increase of the number of iterations. This problem can be solved by increasing the amount of training set data. The predicted deviation value at the specific fault location is approximately 4 km. The prediction accuracy and generalizability of the fault detection model are further improved.

## 4. Fault Isolation Methods

### 4.1. Communication Protocol

In order to realize fault isolation, communication protocol should be established first. There are three main hardware interfaces of an MCU (Microcontroller Unit): UART (Universal Asynchronous Receiver/Transmitter), SPI (Serial Peripheral Interface), and I2C (Inter-Integrated Circuit). This paper selects UART according to the system requirements.

**Figure 11:**
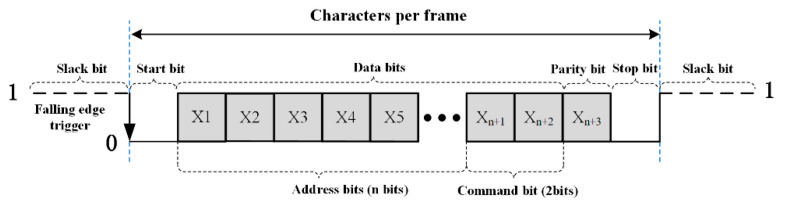
Communication protocol definition.

Combined with the characteristics and application scenarios of BUs, and inspired by serial asynchronous communication protocol, a communication protocol suitable for fault isolation is proposed here, which is shown in Figure 11.

The data bit consists of two parts: the address bits and the command bits. Address bits are mainly used to code and differentiate each BU. The number of address bits is not fixed. The number of address bits can be dynamically adjusted according to the number of nodes in the whole observation network. When the number of communication protocol address bits is n, the maximum controllable number of nodes can reach about 2n. The command bit here is 2 bits, indicating that the number of circuit breakers that can be controlled by each BU is 2, ‘0′ means the switch is turned on, and ‘1′ means the switch is turned off. Parity bits are used to ensure that the entire data are correct. The stop bit ‘0′ is intended to indicate the end of the entire command of the BU, waiting for the next command to be received. Only when the digital current conforms to the communication protocol can the BU switch work, and whether the MCU can correctly sample the current signal is the key of the whole fault isolation system.

### 4.2. Optimal Communication Frequency

For the transmission line system model, the higher the frequency of the sinusoidal signal, the more serious the signal attenuation and distortion. Therefore, when the current square wave is transmitted to a specific BU, the current waveform will be distorted to a certain extent, which will bring uncertainty to the BU sampling unit. When the sampling frequency is high and serious, the original digital signal ‘1′ is sampled at the BU side but becomes ‘0′, which makes the whole communication uncontrollable. This error code will make the whole communication uncontrollable.

**Figure 12:**
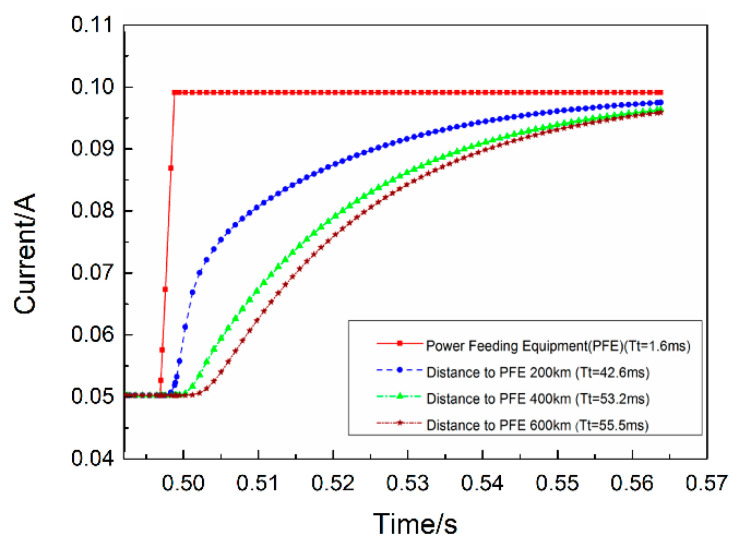
Time response of a branch unit (BU) at different positions of a 600 km cable.

According to the simulation model in Figure 6, the current waveforms of different distances are measured. The power supply terminal is a step current signal of 50–100 mA. From the simulation results showed in Figure 12, it can be seen that the farther away from the shore-based power supply, the more serious the current distortion flowing through the BU sampling resistor, in which *Tt* is the transition time equal to the sum of the rise time and the delay time.

According to the existing simulation data, the algebraic relationship between the maximum transition time *T_t_* and the total length of transmission line *L* is determined by using the MATLAB (MATLAB is a commercial mathematical software produced by MathWorks company in Natick, MA, USA) curve-fitting toolbox. When the polynomial is of second order, the algebraic relationship between *T_t_* and *L* in a 95% confidence interval:

(7) $$T_{t}=0.00015\times L^{2}+0.014\times L-4(L<2000\;\mathrm{km})$$

In order to further increase the accuracy and reliability of sampling, this paper adopts the method of ten-fold frequency sampling: $f_{s}=10\cdot f_{c}$. When considering the distortion degree, the text only considers the influence of the parasitic parameters of the cable on the digital signal. However, in the actual situation, there are still other uncertain factors disturbing the digital current signal, and some disturbance factors are inevitable. For example, the actual transition time *T_t_* will also be increased due to the electric pulse caused by the environment or power supply, estimation error of cable parasitic parameters, software simulation error, curve-fitting error, and response time of electronic components. In order to ensure the accuracy of the sampling accuracy, the sampling frequency f of the switch controller f_s_ must be greater than *T_t_*, where the safety factor S = 2:

(8) $$f_{c}=\frac{1000}{T_{c}}=\frac{1000}{10\cdot (2T_{t})}=\frac{50}{0.00015\times L^{2}+0.014\times L-4}$$

When the transmission distance is within 2000 km, the best communication frequency of the digital current signal can be determined by Equation (8), which not only ensures the high integrity of the signal and improves the reliability of the whole control process, it also shortens the execution time of control instructions in the whole power line transmission process.

### 4.3. Control of BU

**Figure 13:**
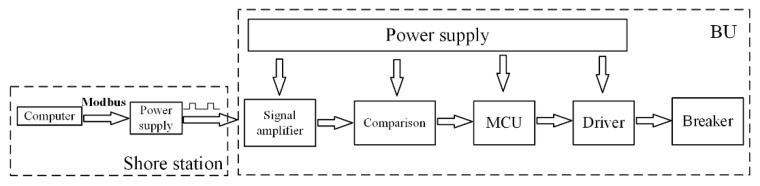
Control method of BU.

The hardware system of a current digital controllable BU mainly includes a power supply and signal acquisition module, control module, and driver module, which is shown in Figure 13.

The SS computer communicates with the SS power supply through Modbus serial communication protocol and sends out the digital current signal of a specific communication frequency. The digital signal reaches the BU through the photoelectric composite cable. Each BU is connected to the backbone cable in series with a voltage regulator and a precision sampling resistor. The voltage regulator acts as the power supply module of the whole BU. The signal amplifier converts the current signal into a voltage signal, and it amplifies the signal to the voltage level that can be processed and identified by the MCU. The analog voltage signal is compared with the reference voltage of the AD conversion chip, and the voltage signal suitable for the MCU is generated. The MCU collects the signals at a certain frequency, combines and recognizes these signals, and decodes them according to the communication protocol. Only when the signal conforms to the communication protocol can the BU switch work.

## 5. Experimental Procedures and Results

### 5.1. Experiment of Fault Detection

**Figure 14:**
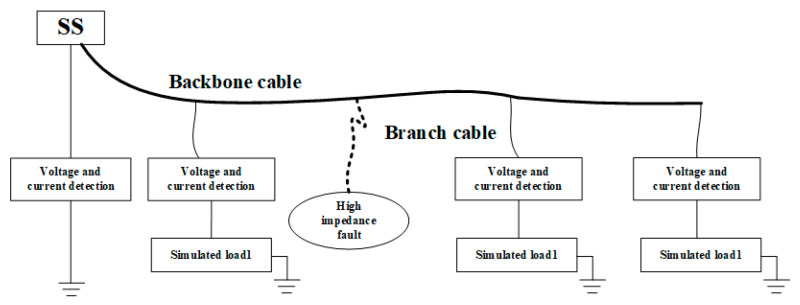
Schematic of fault detection experimental platform.

Under laboratory conditions, the system model is limited by the voltage level of the SS power supply, cable length, cost of experimental equipment, and other constraints. In the experiment, a single power supply and tree topology observation networks are built to verify the proposed fault detection model. The experimental diagram of the experimental platform is shown in Figure 14.

**Figure 15:**
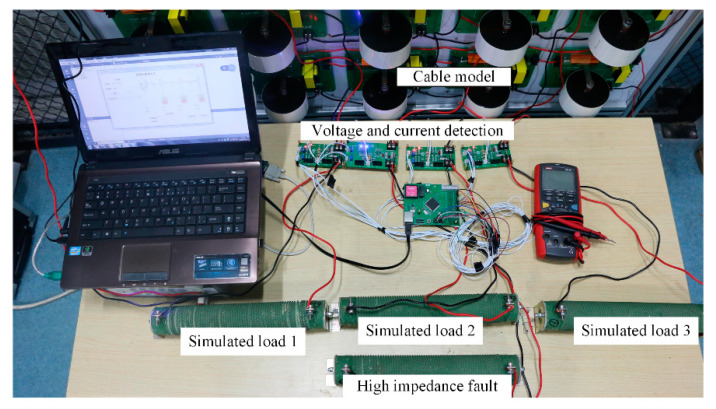
Fault detection experimental platform.

In this laboratory experiment, according to the similarity theory, the voltage level, cable length, and load size are reduced correspondingly compared with the practical observation network. After calculation based on similarity theory, the shore station power supply is reduced from 10 kV to 300 V. The BU and junction box are the load of the system, which are replaced by 50 ohm cement resistance, and the load is set every 60 km. The transmission cable in this experiment is the cable model. The actual observation network may have multiple nodes, but due to the limitation of the experiment scale, only three nodes are set up in the experiment to verify the theory. At the input end of each node, a voltage and current sample are arranged to collect data, which are uploaded to the operating system at the SS through the network port communication of the lower computer STM32. The structure of the laboratory fault detection experiment is shown in Figure 15.

The power supply adopts the constant voltage output of 300 V, the open-circuit fault of the system is assumed to be the disconnection action of the BU, and four sampling circuit boards collect the load and power supply data. PSpice simulation is carried out for each state model of the system. The simulation accuracy of the high-resistance fault is 5 km. Ten different fault resistances are set at the same position, and the resistance value is between 10 and 200. The data from the simulation are used as the training and test samples of the deep learning model.

This experiment is mainly aimed at three working states of the backbone cable: open-circuit fault, high-impedance fault, and normal operation. In the case of a high-impedance fault, the specific location of the fault shall be indicated. Similarly, for an open-circuit fault, the fault detection algorithm must also clearly identify the specific location of the BU causing the open-circuit fault. The relevant experimental results are presented in Table 4.

Four typical cases are extracted from the experimental test sequence for analysis. The system characteristic state vector shown in No. 1 is [0,0,0,0,1,0,0,20]^T^, which means that the first section of the backbone cable of the system has a high-impedance fault (Table 2) and that the actual high-impedance fault point is 20 km away from the beginning of this section. The characteristic vector composed of the voltage and current values of each node is [3.59, 2.05, 102.6, 0.75, 37.4, 0.34, 17.1]^T^. The voltage and current values are used as input in the trained fault detection model, and the prediction result matrix is [0.02,0.01,0.01,0.01,0.88,0.04,0.03,16.8]^T^. The first seven numbers are the results of the softmax classification. The dimension with the highest probability value of the final result is 1, and those of the other dimensions are 0. In the regression prediction of actual fault type, the specific location of the high-impedance fault is predicted to be 16.8 km away from the last branch point when identifying the input characteristics of the fault type of deep learning because the backbone cable accuracy of the fault simulation data is 5 km. A detection error of 3.2 km is identified relative to that of the actual high-impedance fault location. Reducing the simulation accuracy, expanding the fault detection dataset, and further adjusting the parameters of the model can reduce fault detection errors.

The fault type shown in No. 2 is the high-impedance fault at 40 km of the third section of the backbone cable. The detection model can still accurately predict the fault type, and the regression prediction error of the specific location of the fault point is 1.1 km. Serial number 3 is the equivalent open-circuit fault of the second section of the backbone cable caused by the disconnection of BU2. Similarly, serial number 4 is the normal state when the system is running. Each state indicates the sufficient discrimination between features for ensuring that the deep learning model can accurately detect faults.

This paper is aimed at the fault detection of submarine cable. The large disturbance usually comes from a power supply or junction box fault. The output ports of the junction box and shore-based power supply are equipped with over-current and over-voltage protection functions. The proposed method is run to diagnose cable faults on the premise that no large disturbance will occur. The output voltage of the DC power supply used in the submarine observation network has been noise suppressed. The noise is smaller than the useful signal, so the noise has less influence on the diagnostic results. From the results of the experiment, it can be seen that the existence of noise will not affect the judgment of the fault diagnosis results. In addition, if the sensor fails, the accuracy of the proposed fault diagnosis algorithm will be greatly reduced. The sensor needs to be maintained and the fault diagnosis algorithm needs to be run again.

### 5.2. Experiment of Fault Isolation

**Figure 16:**
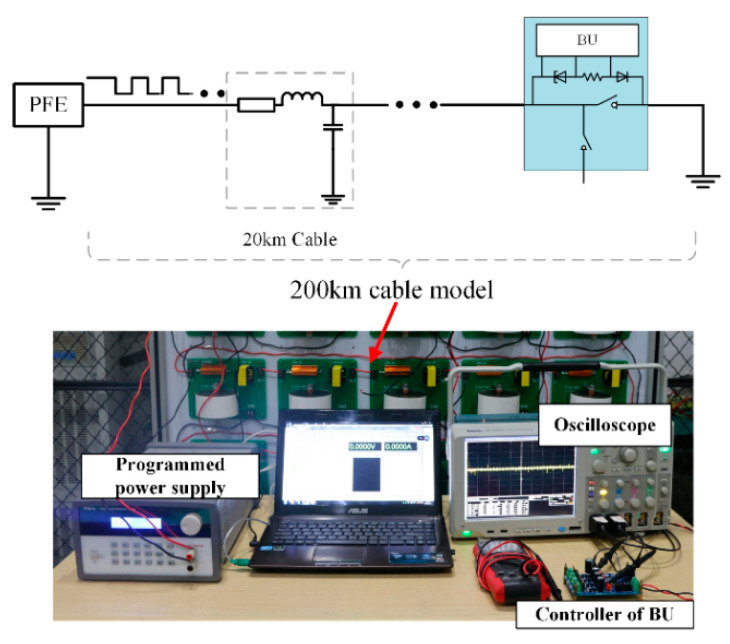
Experiment of fault isolation.

In the fault isolation experiment, the SS power supply sends a digital current of different frequencies to observe whether the BU can reliably carry out the on and off switch. The experimental topology is shown in Figure 16. In the experiment, the BU is installed at 200 km of the cable model. The main difference from the practical system is that the analog cable is used here. The programmable power supply and BU controller are the same as the practical fault isolation system. The ‘0′/‘1′ data current signals are 50 mA/100 mA, respectively.

**Figure 17:**
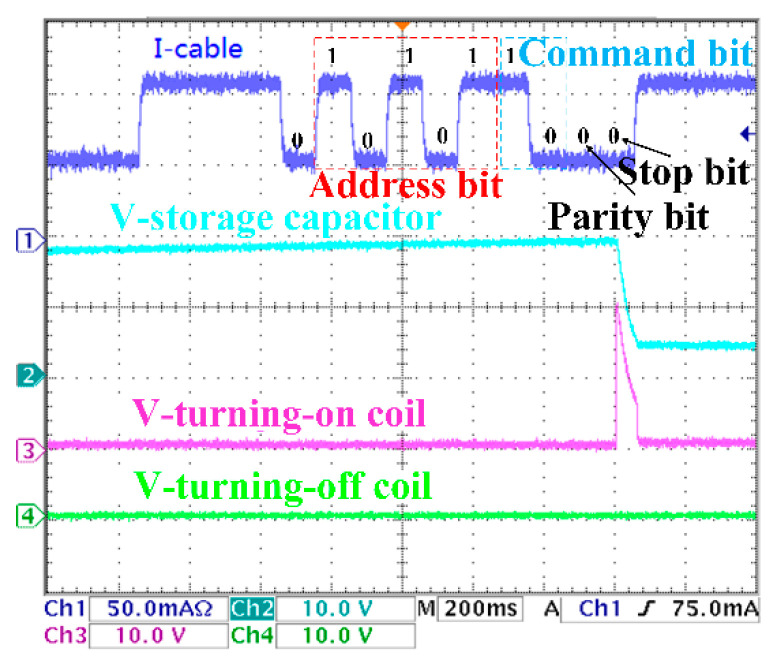
Turning-on command.

**Figure 18:**
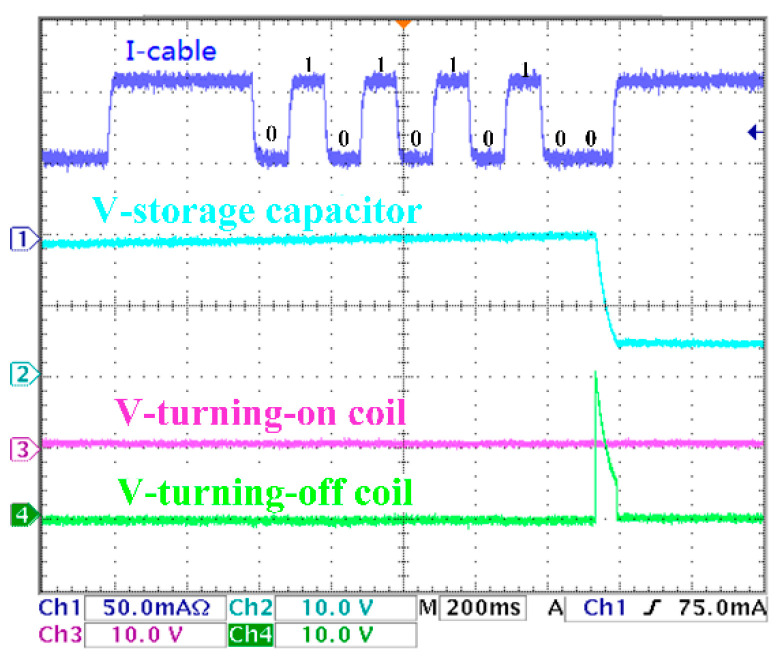
Turning-off command.

**Figure 19:**
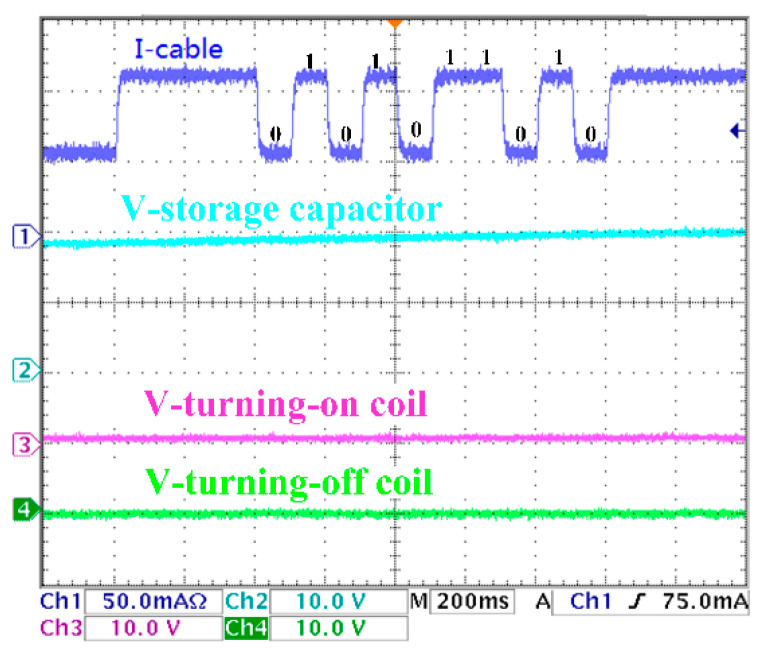
Error parity command.

**Figure 20:**
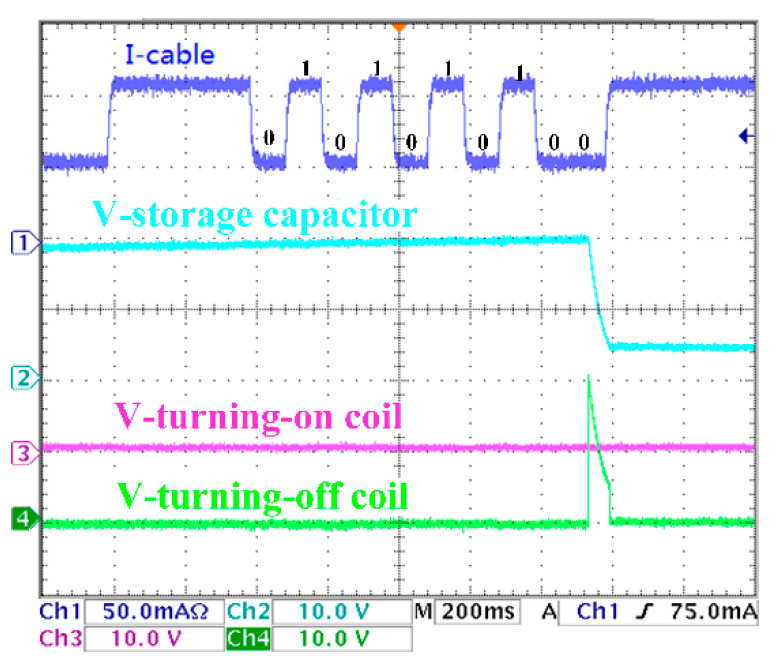
Error address command.

The experimental results are shown in Figure 17, Figure 18, Figure 19 and Figure 20. There are two control coils (turning-on and turning-off coil) in the BU to control the on and off of the switch. According to Equation (8), under the 200 km transmission cable length, the optimal communication rate is $f_{c}=10Hz$; that is, the duration of each digital bit is about 100 ms. Channel 1 (blue) indicates the current signal collected by BU. Channel 2 (cyan) indicates the voltage signal of the voltage storage capacitor. Channel 3 (red) indicates the voltage signal of the turning-on coil. Channel 4 (green) indicates the voltage signal of the turning-off signal. In Figure 17, when the current of the I-Cable is interrupted at the falling edge, the program of the MCU starts. The current value of the backbone cable is collected five times every 100 ms, and the address bit of the BU is obtained as ‘10101′. Furthermore, the command bit is ‘10′, which indicate that the turning-on coil is turned on. The parity bit ‘0′ meets the check requirements. The stop bit is ‘0′, which means that the isolation control procedure is completed, and all input current signals are verified to meet the requirements of communication protocol, and the BU sends control signals to drive the turning-on coil. Figure 18 is similar to Figure 17; the difference is that the command bits change to ‘01′ to drive the turning-off coil. In Figure 19, since the parity bit is ‘1′, which does not meet communication protocol, the BU will not respond. The address bit of Figure 20 is ‘10110′, which does not match the BU address in the experiment, so no misoperation occurs.

**Figure 21:**
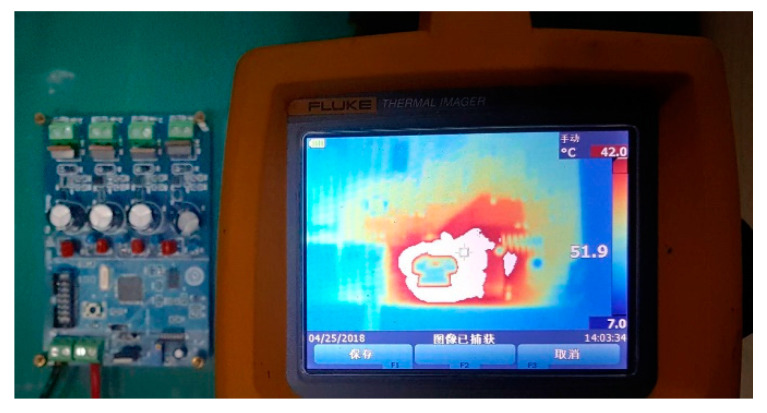
Temperature measurement result of the BU.

In the experimental environment, the reliability of the BU is tested by periodically sending the control commands of turning on/off the switch by the programmable power supply. A simple counting function is realized by software programming. A series of digital current waveforms are sent alternately every 20 s, and the accumulated operation time is 6 h. The actual switching action is consistent with the expectation, and no error occurs. In addition, considering that the actual working environment of the BU is in a closed chamber, and air circulation and heat dissipation conditions are relatively poor, the thermal imager is used to continuously monitor the heating condition of the BU in the durability test in real time. The measurement results are shown in Figure 21.

It can be found from the above figure that the main centralized heating component of the circuit board is the voltage stabilizing tube. Under the working condition of the current value of 100 mA for a long time, the maximum temperature of the component is 52 °C, which is completely within the reasonable range of the component design, which proves the rationality and reliability of the BU.

## 6. Conclusions

With the expansion of the scale of single-line power supply systems, the reliability of submarine observation networks gradually decreases, and the failure of any node and cable will lead to the collapse of entire network systems. To improve the reliability of submarine observation networks, the fault diagnosis and location of transmission lines are studied. On the basis of the characteristic data of the voltage and current of the power supply and underwater nodes, a fault-type diagnosis and location based on DNN is proposed. To verify the feasibility of applying deep learning to fault diagnosis and location, a complex network model with a double SS network topology is adopted in the theoretical research stage. The supervised learning feature engineering of this model is designed in the TensorFlow deep learning framework, and a DNN is built to accelerate the training of the model by using feature preprocessing, the random gradient descent algorithm, and batch normalization. L2 regularization and dropout and other deep learning training strategies are added to prevent over fitting and improve the generalizability of the training model. In terms of fault isolation, a communication protocol suitable for the power supply system of the observation network is proposed, and an experimental platform is built according to this protocol. Experimental results verify the effectiveness of the proposed algorithm for the location and prediction of high-impedance and open-circuit faults, and the feasibility of the fault isolation system has also been verified. Moreover, the proposed methods greatly improve the reliability of undersea observation network systems.

## Figures and Tables

**Table 1:** Training sample eigenvalue.

Training Sample Eigenvalue $x_{}^{\left(i\right)}$		
$x_{1\sim 20}^{\left(i\right)}$	$x_{21\sim 22}^{\left(i\right)}$	$x_{23}^{\left(i\right)}$
10 terminal voltage and current values	2 shore station current values	2 shore station current product values

**Table 2:** Calibration value of training sample.

Training Sample Eigenvalue $y_{}^{\left(i\right)}$			
$y_{1}^{\left(i\right)}$	$y_{2\sim 13}^{\left(i\right)}$	$y_{14\sim 27}^{\left(i\right)}$	$y_{28}^{\left(i\right)}$
Normal/fault 1/0	BU Open-circuit fault/normal 1/0	High-impedance fault/normal 1/0	Fault location

**Table 3:** Hyperparameters of this model.

Hyperparameter	Function of Parameter
Relative = 100	Regression of loss function and classification weight of softmax
DataSplitRate = 0.8	Data partition ratio of training set and test set
TrainingSteps = 20,000	Number of training iterations
Learning_rate = 0.001	Learning rate
Keep_prob = 0.8	Dropout regularization coefficient
Layer_dimension = [22, 20, 40, 28]	Number of hidden layers and number of nerve units in each layer

**Table 4:** Experimental results of fault detection.

	Test Set							Calibration/Prediction							
No.	IS	I#1	V#1	I#2	V#2	I#3	V#3	N	K1	K2	K3	G1	G2	G3	D
1	3.59	2.05	102	0.75	37.4	0.34	17.1	0	0	0	0	1	0	0	20
								0.02	0.01	0.01	0.01	0.88	0.04	0.03	16.8
2	3.24	2.10	105	0.75	37.8	0.33	16.4	0	0	0	0	0	0	1	40
								0.01	0.02	0.01	0.02	0.01	0.03	0.90	38.9
3	2.73	2.73	136	0	0	0	0	0	0	1	0	0	0	0	0
								0.01	0.01	0.98	0.01	0.00	0.00	0.00	1.4
4	3.28	2.16	105	0.82	38.6	0.82	17.6	1	0	0	0	0	0	0	0
								0.91	0.01	0.01	0.01	0.02	0.02	0.02	0.7

## Appendix A

**Algorithm A1.** Script of function add_layer.
def add_layer (inputs, in_size, out_size, n_layer, activation_function = None, BN_able = True, train_phase = True): layer_name = ‘layer%s’ % n_layer with tf.name_scope (layer_name): with tf.name_scope (‘weights’): Weights = tf.Variable (tf.random_normal ([in_size, out_size]), name = ‘W’) tf.summary.histogram (‘Histogram’, Weights) with tf.name_scope (‘biases’): biases = tf.Variable (tf.zeros ([1, out_size]) + 0.1, name = ‘b’) tf.summary.histogram (‘Histogram’, biases) with tf.name_scope (‘Wx_plus_BN_before’): Wx_plus_b = tf.matmul (inputs, Weights) + biases Wx_plus_b = tf.nn.dropout (Wx_plus_b, keep_prob) if BN_able: with tf.variable_scope (layer_name): fc_mean, fc_var = tf.nn.moments (#Get the mean value and variance value of each feature (dimension) Wx_plus_b, axes = [0], # the dimension you want to normalize, here [0] for batch ) with tf.name_scope (‘scale’): scale = tf.Variable (tf.ones ([out_size]), name = ‘scale’) with tf.name_scope (‘shift’): shift = tf.Variable (tf.zeros ([out_size]), name = ‘shift’) epsilon = 0.001 ema = tf.train.ExponentialMovingAverage ( decay = 0.5) def mean_var_with_update (): ema_apply_op = ema.apply ([fc_mean, fc_var]) with tf.control_dependencies ([ema_apply_op]): return tf.identity (fc_mean), tf.identity (fc_var) mean, var = tf.cond (tf.equal (train_phase, True), mean_var_with_update, lambda: (ema.average (fc_mean), ema.average (fc_var))) with tf.name_scope (‘Wx_plus_BN_after’): Wx_plus_b = tf.nn.batch_normalization (Wx_plus_b, mean, var, scale, shift, epsilon) if activation_function is None: outputs = Wx_plus_b else: outputs = activation_function (Wx_plus_b) tf.summary.histogram (‘outputs/Histogram’, outputs) return outputs

**Algorithm A2.** Script of cost functions.
cross_entropy = tf.reduce_mean (-tf.reduce_sum(relative * ys1 * tf.log (tf.clip_by_value (predict1, 1e-10, 1.0)), reduction_indices = 1)) mean_squared = tf.losses.mean_squared_error (output2, ys2) with tf.name_scope (‘loss’): loss = cross_entropy + mean_squared tf.summary.scalar (‘loss’, loss) with tf.name_scope (‘train’): train_op = tf.train.AdamOptimizer (learning_rate = Learning_rate).minimize (loss) with tf.Session () as sess: merged = tf.summary.merge_all () # summary writer goes in here train_writer = tf.summary.FileWriter (“logs/train”, sess.graph) test_writer = tf.summary.FileWriter (“logs/test”, sess.graph) init = tf.global_variables_initializer () sess.run (init) for i in range (TrainingSteps): sess.run (train_op, feed_dict = {xs: trainset, ys: trainlabels, keep_prob: Keep_prob, train_phase: True}) if i % RecordSteps == 0: print (“After %d training step(s):” % i) print (“TrainingSet Cost Value:”, sess.run (loss, feed_dict = {xs: trainset, ys: trainlabels, keep_prob: 1, train_phase: True})) print (“TrainingSet Accuracy:”, compute_accuracy (trainset, trainlabels, True)) print (“TestSet Cost Value:”, sess.run (loss, feed_dict = {xs: testset, ys: testlabels, keep_prob: 1, train_phase: False})) print (“TestSet Accuracy:”, compute_accuracy (testset, testlabels, False)) print () train_result = sess.run (merged, feed_dict = {xs: trainset, ys: trainlabels, keep_prob: 1, train_phase: True}) test_result = sess.run (merged, feed_dict = {xs: testset, ys: testlabels, keep_prob: 1, train_phase: False}) train_writer.add_summary (train_result, i) test_writer.add_summary (test_result, i)
